# Differential Association of Wheat and Rice Consumption With Overweight/Obesity in Chinese Adults: China Health and Nutrition Survey 1991–2015

**DOI:** 10.3389/fnut.2022.808301

**Published:** 2022-06-06

**Authors:** Jiguo Zhang, Zhihong Wang, Wenwen Du, Feifei Huang, Bing Zhang, Huijun Wang

**Affiliations:** Key Laboratory of Trace Element Nutrition of National Health Commission, Department of Public Nutrition, National Institute for Nutrition and Health, Chinese Center for Disease Control and Prevention, Beijing, China

**Keywords:** wheat, rice, overweight, obesity, adults

## Abstract

Wheat and rice are the main staple foods in China and likely have a major influence on health. This analysis examined the potential association between wheat and rice consumption and the risk of overweight/obesity in Chinese adults. We used data collected in the China Health and Nutrition Survey (CHNS) from 1991 to 2015. Adults aged 18–80 years old (*n* = 11,503) were included in the present analysis, for whom questionnaires and anthropometric data were collected during at least two waves. We constructed three-level mixed-effect linear regression models to estimate body mass index (BMI) in relation to wheat and rice intakes and performed three-level mixed-effect logistic regression models to assess the risk of overweight/obesity. Women showed significant BMI increases of 0.14 (95% CI: 0.04, 0.24) from a higher intake of wheat but not from a higher intake of rice when adjusted for all potential confounders. Comparing the highest quartiles of intake of wheat with non-consumers in men and women, odds ratios (ORs; 95% CI) of overweight/obesity were 1.45 (1.15, 1.85) and 1.26 (1.00, 1.60), respectively. In men, there was an inverse association with the risk of overweight/obesity in the comparison of the highest quartiles of intake of rice (OR: 0.73; 95% CI: 0.55, 0.96) and non-consumers when adjusted for all potential confounders. Higher intake of wheat was positively associated with the risk of overweight/obesity among Chinese adults. Further, there was an inverse association between rice intake with overweight/obesity in Chinese men but not in women.

## Introduction

Overweight and obesity have become major public health problems in both developing and developed countries (1). The prevalence has increased substantially in recent decades around the world, including in China (2–4). The 2020 Report on the Nutrition and Chronic Disease Status of Chinese Residents showed that more than half of adults are overweight or obese. Nowadays, the net population of obese Chinese individuals is among the highest in the world (5). Obesity is not only a chronic condition but also a risk factor that leads to multiple non-communicable diseases (NCDs), such as type 2 diabetes, cardiovascular disease, hypertension and stroke, several types of cancers, as well as poor mental health (6). It has been estimated that a medical cost of ¥418 billion (~US$61 billion), accounting for about 22% of total national medical costs, attributed to overweight and obesity in China by 2030 (7). China is facing the consequences of obesity-related health problems.

Some studies have shown the association of obesity with rice-based and wheat-based dietary patterns, although the results are not consistent (8–15). It is difficult to tell the exact mechanism underlying the associations due to the different components of food items. However, one common characteristic of the patterns was a high intake of wheat or rice. Because these studies focused on patterns of food group intake rather than on intakes of single food groups, conclusions about whether wheat and rice have an association with obesity cannot be inferred directly from these findings. Wheat and rice are the main staple foods in China, which contribute to about half of the total energy intake (16). Considering the frequency and amount of consumption, wheat and rice are likely to have a major influence on health, either good or adverse. To our knowledge, studies to determine the potentially different health influences of wheat and rice are limited, and the association has not been well-investigated in large populations. The present analysis was to examine the prospective association between wheat and rice consumption and the risk of overweight/obesity in Chinese adults.

## Materials and Methods

### Study Population

We used data collected in the China Health and Nutrition Survey (CHNS), an ongoing large-scale, longitudinal, household-based survey of 10 waves, which was conducted in 1989, 1991, 1993, 1997, 2000, 2004, 2006, 2009, 2011, and 2015 (17, 18). The CHNS used a multistage random-cluster process to draw the sample in nine provinces from northeast to southwest: Heilongjiang, Liaoning, Jiangsu, Shandong, Henan, Hubei, Hunan, Guizhou, and Guangxi. We added three megacities (Beijing, Chongqing, and Shanghai) in 2011 and three new provinces (Shaanxi, Yunnan, and Zhejiang) in 2015. Finally, the CHNS covered 15 provinces that varied in demography, geography, economic development, and public resources. In each province, two cities and four counties were selected by income (low, middle, and high). A total of four communities from each city/county were selected randomly; in each community, 20 households were randomly selected and followed in 9 subsequent waves. All individuals within a household were interviewed. In some cases, new households were recruited to substitute for those who migrated out of the community for different reasons. Such sampling reflects the hierarchical data structure: measurement occasions (level 1) for individuals (level 2) nested in communities (level 3).

Our analysis used the nine waves of survey data between 1991 and 2015. Adults aged 18–80 years were included in the present analysis, for whom questionnaires and anthropometric data were collected during at least two waves. Final analyses were conducted among individuals with complete data on dietary, anthropometric, demographic, socioeconomic, and other lifestyle factors. Therefore, the total number of participants and observations were 11,503 and 47,322, respectively. The study was approved by the Institutional Review committees of the University of North Carolina at Chapel Hill and National Institute for Nutrition and Health, Chinese Center for Disease Control and Prevention. Participants provided their written, informed consent.

### Dietary Measurements

Each wave of the survey assessed dietary intake using three consecutive 24-h dietary recalls (2 weekdays and 1 weekend day). Interviewers were trained to use standard forms to administer the dietary recalls in household interviews. The participants were asked to report the kinds and amounts of food and beverage items (measured in grams) that they ate both at home and away from home during a 24-h period. We used the average intake of wheat and rice from the three consecutive 24-h periods for each individual. Wheat included noodles, buns, pancakes, and other products. Rice included round-grained rice, long-grained rice, glutinous rice, and other products.

### Other Relevant Variables

All waves of the CHNS have obtained clinical, anthropometric, and all other individual data from each household member. A general questionnaire collected participant's age, education (low, medium and high), living area (urban and rural), smoking status (current, former, or never smoker), alcohol consumption (yes or no), physical activity (four domains: occupational, household chore, leisure time, and transportation activities), and annual household income per family member (low, medium, and high). Well-trained health workers who followed a reference protocol recommended by the WHO collected anthropometrical measurements. Body mass index (BMI) was calculated using height and weight measurements. In the present study, overweight/obesity was defined as a BMI ≥ 24 kg/m^2^ (19).

### Statistical Analyses

We categorized participants into non-consumers and quartiles of wheat and rice. We used chi-square tests (categorical variables) and analysis of variance and non-parametric Kruskal-Wallis tests (continuous variables) to compare the characteristics of participants across the wheat and rice intake levels. We constructed three-level mixed-effect linear regression models (xtmixed in STATA) to estimate BMI in relation to wheat and rice intakes and performed three-level mixed-effect logistic regression models (gllamm in STATA) to assess the risk of overweight/obesity. We calculated regression coefficients (95% CIs) and odds ratios (ORs) (95% CIs), respectively. We conducted all analyses separately for the intakes of rice and wheat and stratified them by gender. We constructed two sequential models. Model 1 adjusted for age at baseline, living area, individual income, education level, physical activity, smoking status, and alcohol consumption. Model 2 additionally adjusted for total energy intake and intake of fruit, vegetables, and meat. Moreover, we evaluated linear trends by assigning participants the median value for quartiles of intake of wheat and rice among consumers, and we entered this variable as a continuous term in the regression model.

All statistical tests were two-tailed, and we regarded differences as significant at *P* < 0.05. For all analyses, we used SAS (Version 9.4, SAS Institute Inc., Cary, NC, United States) and Stata/SE (STATA, Version 15, StataCorp, College Station, TX, United States).

## Results

Table 1 presents the characteristics of participants across five levels of wheat and rice consumption by gender in CHNS 1991. It is notable that wheat consumption was significantly inversely associated with rice consumption in both men and women. Participants in the top quartile of the wheat and rice consumption had a higher intake of energy, higher energy from carbohydrates, lower education level, were more physically active, and were less likely to live in urban areas than those in the lowest quartile. Wheat consumption was positively associated with BMI but inversely associated with fruit and vegetable intake. In contrast, rice consumption was inversely associated with BMI but positively associated with fruit and vegetable intake.

**Table 1:** Characteristics of participants across five levels of wheat and rice consumption by gender; China Health and Nutrition Survey (CHNS) 1991^a^.

**Characteristics**	**Men**					***P***	**Women**					***p***
	**Non-consumers**	**Q1**	**Q2**	**Q3**	**Q4**		**Non-consumers**	**Q1**	**Q2**	**Q3**	**Q4**	
**Wheat**												
No. of participants	894	407	410	406	407		868	410	389	391	395	
Wheat intake (g/day)^b^	0.0	54.3 ± 19.8	118.4 ± 20.0	232.3 ± 53.4	529.0 ± 119.8		0.0	45.9 ± 16.5	104.3 ± 18.1	216.2 ± 57.3	496.3 ± 126.1	
Age (years)	40.1 ± 13.4	39.2 ± 14.3	39.8 ± 14.4	40.0 ± 14.1	39.1 ± 13.2	0.69	40.3 ± 13.6	39.8 ± 14.2	38.4 ± 14.2	39.2 ± 13.2	38.9 ± 13.6	0.15
Urban (%)	14.9	38.3	45.6	37.0	26.0	<0.0001	15.9	43.2	42.9	39.1	20.0	<0.0001
Income level (high) (%)	24.4	40.0	42.0	43.8	27.0	<0.0001	26.8	40.7	42.7	40.7	23.5	0.07
Education (high) (%)	11.5	22.8	23.2	24.9	17.4	<0.0001	6.0	17.8	21.6	17.9	8.9	<0.0001
Current smoker (%)	71.9	66.3	70.7	72.4	73.7	0.34	3.7	4.4	3.9	4.9	4.3	0.46
Alcohol consumption (%)	63.3	67.3	65.8	68.2	65.6	0.21	12.0	11.9	11.3	15.6	12.4	0.35
Physical activity (MET hours/week)	487.5 ± 234.6	378.8 ± 253.8	315.4 ± 226.0	353.6 ± 248.2	430.7 ± 236.3	<0.0001	553.7 ± 261.6	419.2 ± 271.2	357.9 ± 256.9	415.2 ± 289.7	517.1 ± 275.3	<0.0001
BMI (kg/m^2^)	20.3 ± 1.7	20.6 ± 1.6	20.6 ± 1.7	20.8 ± 1.7	21.1 ± 1.6	<0.0001	20.4 ± 1.7	20.5 ± 1.9	20.7 ± 1.7	21.0 ± 1.7	21.2 ± 1.7	<0.0001
Total energy (kcal/day)	2,386.5 ± 757.8	2,489.6 ± 678.0	2,611.4 ± 700.3	2,786.2 ± 775.7	2,956.8 ± 643.2	<0.0001	2,097.1 ± 630.2	2,080.0 ± 617.4	2,281.4 ± 580.7	2,376.8 ± 603.9	2,630.3 ± 503.0	<0.0001
Carbohydrate (% of energy)	60.2 ± 14.8	58.3 ± 12.9	58.5 ± 11.3	61.9 ± 11.6	69.2 ± 9.1	<0.0001	62.0 ± 13.6	59.2 ± 10.8	61.3 ± 11.2	64.1 ± 10.8	70.4 ± 9.1	<0.0001
Rice intake (g/day)^c^	500.0 (400.0, 616.7)	416.7 (333.3, 516.7)	350.0 (283.3, 433.3)	296.7 (116.7, 416.7)	0.0 (0.0, 100.0)	<0.0001	450.0 (355.0, 550.0)	350.0 (267.8, 450.0)	300.0 (250.0, 400.0)	214.3 (83.3, 333.3)	0.0 (0.0, 83.3)	<0.0001
Meat (g/day)	50.0 (0.0, 100.0)	71.4 (23.3, 125.0)	80.0 (31.7, 133.3)	50.0 (0.0, 100.0)	0.0 (0.0, 62.5)	<0.0001	33.3 (0.0, 83.3)	66.7 (23.3, 108.3)	58.3 (16.7, 102.3)	33.3 (0.0, 75.0)	0.0 (0.0, 41.7)	<0.0001
Fruit and vegetables (g/day)	400.0 (280.0, 541.7)	333.3 (233.3, 450.0)	326.7 (233.3, 433.3)	283.3 (200.0, 408.3)	266.7 (166.7, 403.3)	<0.0001	365.2 (250.0, 516.7)	300.8 (216.7, 408.3)	291.7 (208.3, 408.3)	283.3 (183.3, 400.0)	258.3 (178.3, 350.0)	<0.0001
**Rice**												
No. of participants	379	505	546	559	535		357	497	550	507	542	
Rice intake (g/day)^b^	0.0	167.7 ± 85.6	356.1 ± 36.0	474.6 ± 35.2	659.8 ± 95.6		0.0	136.8 ± 68.5	305.2 ± 33.4	417.2 ± 32.6	584.8 ± 93.1	
Age (years)	39.9 ± 13.7	41.8 ± 15.1	40.3 ± 14.0	38.9 ± 13.8	37.7 ± 11.9	<0.0001	39.1 ± 13.7	39.6 ± 14.5	40.4 ± 15.2	39.7 ± 13.5	38.6 ± 11.5	0.26
Urban (%)	18.5	46.7	46.0	22.4	9.3	<0.0001	16.2	45.1	47.8	24.3	8.5	<0.0001
Income level (high) (%)	19.3	46.3	43.8	33.4	20.2	0.00	14.6	46.5	47.1	34.7	18.4	0.00
Education (high) (%)	12.4	27.5	25.3	15.7	9.5	<0.0001	5.9	20.7	20.4	11.0	4.1	<0.0001
Current smoker (%)	72.8	73.1	70.3	68.3	72.2	0.37	5.3	5.2	4.2	3.0	3.3	0.03
Alcohol consumption (%)	62.5	67.7	64.8	64.9	66.9	0.47	9.0	16.7	11.4	11.0	13.5	0.73
Physical activity (MET hours/week)	489.2 ± 230.0	310.5 ± 244.0	308.3 ± 221.5	437.5 ± 248.2	529.1 ± 205.7	<0.0001	540.0 ± 269.4	357.3 ± 262.5	358.7 ± 258.5	489.7 ± 276.0	631.7 ± 224.2	<0.0001
BMI (kg/m^2^)	21.0 ± 1.6	20.9 ± 1.7	20.5 ± 1.7	20.4 ± 1.6	20.4 ± 1.6	<0.0001	21.1 ± 1.7	21.0 ± 1.8	20.6 ± 1.9	20.4 ± 1.7	20.5 ± 1.7	<0.0001
Total energy (kcal/day)	2,469.0 ± 859.1	2,448.4 ± 700.6	2,514.8 ± 690.9	2,580.8 ± 686.7	2,923.6 ± 743.6	<0.0001	2,288.0 ± 770.2	2,113.5 ± 579.4	2,114.7 ± 599.8	2,224.7 ± 565.1	2,528.7 ± 560.4	<0.0001
Carbohydrate (% of energy)	63.0 ± 18.0	59.6 ± 13.4	56.9 ± 11.6	61.8 ± 11.2	65.9 ± 10.2	<0.0001	66.2 ± 16.6	61.8 ± 12.1	58.7 ± 10.9	62.5 ± 10.0	67.4 ± 9.9	<0.0001
Wheat intake (g/day)^c^	433.3 (133.3, 583.3)	200.0 (100.0, 384.6)	100.0 (33.3, 150.0)	30.8 (0.0, 100.0)	0.0 (0.0, 33.3)	<0.0001	400.0 (225.0, 558.3)	200.0 (100.0, 350.0)	73.2 (25.0, 133.3)	0.0 (0.0, 83.3)	0.0 (0.0, 33.3)	<0.0001
Meat (g/day)	0.0 (0.0, 50.0)	50.0 (0.0, 100.0)	83.3 (33.3, 133.3)	58.3 (0.0, 116.7)	33.3 (0.0, 91.7)	<0.0001	0.0 (0.0, 33.3)	35.7 (0.0, 76.9)	66.7 (25.0, 116.7)	50.0 (0.0, 93.3)	16.7 (0.0, 67.3)	<0.0001
Fruit and vegetables (g/day)	250.0 (166.7, 383.3)	270.8 (200.0, 400.0)	316.7 (233.3, 416.7)	365.0 (258.3, 483.3)	450.0 (307.7, 591.7)	<0.0001	232.1 (150.0, 333.3)	266.7 (166.7, 383.3)	291.7 (216.7, 400.0)	333.3 (233.3, 450.0)	416.7 (300.0, 550.0)	<0.0001

a *MET hours, metabolic equivalent hours; BMI, body mass index. Differences across five levels were obtained by using ANOVA, the Kruskal-Wallis test, or the chi-square test*.

b *Mean ± SD (all such values)*.

c *Median (range) (all such values)*.

Table 2 shows the longitudinal association between levels of wheat and rice consumption and BMI stratified by gender. Women showed significant BMI increases of 0.14 (95% CI: 0.04, 0.24) from a higher intake of wheat but not from a higher intake of rice when adjusted for all potential confounders. In men, we observed no significant association between the consumption of wheat and rice with BMI.

**Table 2:** Regression coefficients (95% CI) of body mass index (BMI) according to the level of intake of wheat and rice among Chinese adults, CHNS^a^^c^.

	**Non-consumers**	**Q1**	**Q2**	**Q3**	**Q4**	***P*-trend^b^**
**Men**						
**Wheat**						
Model 1	0.00 (ref)	−0.03 (−0.10, 0.04)	−0.03 (−0.10, 0.05)	0.04 (−0.04, 0.12)	0.10 (0.00, 0.20)	0.55
Model 2	0.00 (ref)	−0.03 (−0.10, 0.04)	−0.03 (−0.10, 0.04)	0.02 (−0.05, 0.10)	0.06 (−0.04, 0.17)	0.75
**Rice**						
Model 1	0.00 (ref)	−0.04 (−0.13, 0.06)	0.04 (−0.08, 0.15)	0.00 (−0.11, 0.12)	0.05 (−0.07, 0.17)	0.06
Model 2	0.00 (ref)	−0.04 (−0.14, 0.06)	0.02 (−0.09, 0.13)	−0.02 (−0.14, 0.09)	0.00 (−0.12, 0.12)	0.47
**Women**						
**Wheat**						
Model 1	0.00 (ref)	0.02 (−0.05, 0.09)	0.10 (0.03, 0.16)^**^	0.10 (0.02, 0.18)^*^	0.15 (0.06, 0.25)^**^	0.02
Model 2	0.00 (ref)	0.02 (−0.05, 0.09)	0.09 (0.02, 0.16)^*^	0.09 (0.01, 0.17)^*^	0.14 (0.04, 0.24)^**^	0.06
**Rice**						
Model 1	0.00 (ref)	0.03 (−0.07, 0.13)	−0.00 (−0.11, 0.11)	−0.01 (−0.12, 0.10)	−0.01 (−0.13, 0.10)	0.51
Model 2	0.00 (ref)	0.03 (−0.07, 0.12)	−0.01 (−0.12, 0.10)	−0.03 (−0.14, 0.09)	−0.04 (−0.16, 0.08)	0.21

* *P < 0.05*,

** *P < 0.01*.

a *All of the models were constructed using three-level mixed-effect linear regression*.

b *P-trend was calculated across the quartiles of wheat and rice among consumers, and this variable was entered as a continuous term in the regression models*.

c *Model 1 adjusted for age at baseline, living area, individual income, education level, physical activity, smoking status, and alcohol consumption. Model 2 additionally adjusted for total energy intake and intake of fruit, vegetables, and meat*.

As Table 3 shows, there was a positive association between the risk of overweight/obesity and wheat consumption. Comparing the highest quartiles of intake of wheat with non-consumers in men and women, ORs (95% CI) of overweight/obesity were 1.45 (1.15, 1.85) and 1.26 (1.00, 1.60), respectively. In men, we found an inverse association with the risk of overweight/obesity in the comparison of the highest quartiles of intake of rice (OR: 0.73; 95% CI: 0.55, 0.96) and non-consumers when adjusted for all potential confounders. We observed no significant association between intake of rice and risk of overweight/obesity in women.

**Table 3:** Odds ratios (ORs; 95% CI) of overweight/obesity across levels of wheat and rice consumption among Chinese adults, CHNS^a^^c^.

	**Non-consumers**	**Q1**	**Q2**	**Q3**	**Q4**	***P*-trend^b^**
**Men**						
**Wheat**						
Model 1	1.00 (ref)	1.00 (0.83, 1.21)	1.17 (0.96, 1.43)	1.18 (0.96, 1.45)	1.46 (1.15, 1.85)^**^	0.00
Model 2	1.00 (ref)	1.01 (0.83, 1.22)	1.16 (0.95, 1.41)	1.17 (0.96, 1.44)	1.45 (1.15, 1.85)^**^	0.00
**Rice**						
Model 1	1.00 (ref)	0.85 (0.68, 1.07)	0.82 (0.63, 1.06)	0.78 (0.60, 1.02)	0.81 (0.61, 1.07)	0.20
Model 2	1.00 (ref)	0.84 (0.67, 1.07)	0.78 (0.60, 1.01)	0.72 (0.55, 0.95)^*^	0.73 (0.55, 0.96)^*^	0.02
**Women**						
**Wheat**						
Model 1	1.00 (ref)	1.09 (0.91, 1.31)	1.14 (0.95, 1.37)	1.28 (1.05, 1.55)^*^	1.31 (1.04, 1.65)^*^	0.02
Model 2	1.00 (ref)	1.09 (0.91, 1.31)	1.14 (0.95, 1.37)	1.26 (1.03, 1.53)^*^	1.26 (1.00, 1.60)^*^	0.06
**Rice**						
Model 1	1.00 (ref)	1.01 (0.79, 1.28)	1.04 (0.79, 1.36)	1.00 (0.76, 1.32)	1.03 (0.78, 1.36)	0.84
Model 2	1.00 (ref)	1.01 (0.80, 1.28)	1.02 (0.78, 1.34)	0.97 (0.74, 1.28)	0.97 (0.73, 1.29)	0.44

* *P < 0.05*,

** *P < 0.01*.

a *All of the models were constructed using three-level mixed-effect logistic regression*.

b *P-trend was calculated across the quartiles of wheat and rice among consumers, and this variable was entered as a continuous term in the regression models*.

c *Model 1 adjusted for age at baseline, living area, individual income, education level, physical activity, smoking status, and alcohol consumption. Model 2 additionally adjusted for total energy intake and intakes of fruit, vegetables, and meat*.

## Discussion

In this large-scale, prospective cohort study of the Chinese population, we found that a high intake of wheat was positively associated with an increased BMI measurement (women only) and risk of overweight/obesity among Chinese adults aged 18–80. Moreover, we observed an inverse association between rice intake with overweight/obesity in men independent of intake of fruit, vegetables, and meat, energy intake, and lifestyle factors. To the best of our knowledge, the present study is the first that investigates the longitudinal association of cereals intake with BMI and overweight/obesity in Chinese adults with an emphasis on the potentially different roles of wheat and rice.

Within China, the Yangtze River splits the wheat-growing north from the rice-growing south. For generations, northern China has grown wheat and southern China has grown rice (20). Because of the agricultural difference, people living in northern China are more likely to eat staple foods made from wheat, such as noodles, flat cakes, and steamed buns. In the south of China, people prefer to eat rice as a staple food with dishes. Traditionally, then, there is a northern dietary pattern of high intake of wheat and a southern dietary pattern characterized by a high intake of rice, vegetables, and meat (15, 21). Previous studies have revealed the differences in the prevalence of obesity and associated metabolic abnormalities among Chinese people living in the north and south (22, 23).

Because of rapid economic and social development in China, the wheat-based pattern has become more prominent. It includes many energy-dense foods, such as cakes/cookies/pastries, deep-fried wheat, and instant noodles, which may affect diet quality (24). The positive association between wheat intake and overweight/obesity in our study is consistent with previous studies of wheat-based dietary patterns. In Chinese young women, a dietary pattern of high intake of wheat, other cereals, and tubers are positively associated with general and abdominal obesity (8). This is similar to the noodle dietary pattern in Korean women that were associated with an increased risk of abdominal obesity (13). A follow-up study shows that daily intake of udon noodles may increase the risk of abdominal obesity in Japanese urban populations (25). Another ecological study suggests that wheat availability is an independent predictor of obesity prevalence both worldwide and in regions of Africa, America, and Asia (26). Recently, the Chinese Longitudinal Healthy Longevity Survey showed that older men in China who consumed wheat as their staple food had greater WCs and higher BMIs than those who consumed rice (27). However, a review concludes that wheat consumption cannot be linked to the increased prevalence of obesity in the general population (28). The inconsistency could be partially due to the different effects of whole and refined wheat (29, 30). Whole wheat products are rich in fiber, micronutrients, and minerals while refined wheat contains, practically, only carbohydrates, which are less beneficial nutritionally (26, 31). In China, daily whole-grain intake was estimated to have decreased from 91 g in 1982 to 14.6 g in 2012, and it is likely to have continued to decline since 2012 (32). Thus, the observed correlation in our study may be a result of refined wheat consumption.

The mechanism underlying the positive association is unclear. One explanation could be wheat gluten (33). Existing animal studies have shown that gluten intake can contribute to an acceleration of body weight gain that results from a reduction in energetic expenditure, as well as the beneficial effects of gluten-free diets in reducing adiposity gain (34, 35). Another possible cause could be a lower intake of micronutrients; that is, high wheat consumption is a low-nutrient diet (8). Micronutrient deficiencies may be important contributors to obesity because micronutrients, such as iron, vitamin C, and vitamin A, may play an important role in fat deposition and the pathogenesis of obesity (36, 37).

Interestingly, a higher intake of rice was inversely associated with the risk of overweight/obesity only in men. This is consistent with a 7-year longitudinal study in the Jiangsu province of China, which revealed that rice intake was inversely associated with weight gain (38). It is also consistent with studies of rice-based dietary patterns (8–10, 14, 15). However, there is still dispute about the role of a rice-based dietary pattern in preventing obesity in Asian countries (11–13). In contrast with our results, a study of Japanese workers showed the consumption of white rice was positively correlated with the risk of a 1-year body weight gain of 3 kg or more (39). Further, a study of an Iranian population reported a non-significant association between intake of rice and central obesity (40). This discrepancy could be explained by the different sources of rice. In China, most of the rice consumed is white rice, which has a high glycemic index (GI) and is a predominant contributor to the dietary glycemic load (41, 42). Some studies show that different types of rice (white vs. brown rice) result in different glycemic responses, and their consumption may affect dietary management of obesity (39, 43). The protective role of rice intake may be owing to the up-regulation of lipolysis and the down-regulation of lipogenesis by rice protein, which may improve body weight and adiposity (44), so it could reduce the negative effect of high GI rice (40). In addition, Abubakar et al. demonstrated that germination and amylose in rice may have beneficial properties in reducing the burden of obesity (45). Thirdly, rice consumption was positively associated with fruit and vegetable intake in our study, which is known to be associated with a reduced risk of measures of adiposity, including overweight/obesity, abdominal obesity, or weight gain, respectively (30). Nevertheless, the inverse rice and overweight/obesity association remained significant after fruit and vegetable adjustment.

The strengths of the present study include its large size, its prospective design, and the wide variation in wheat and rice, which made it possible to detect weak associations and to perform stratified analyses by only losing a small proportion of statistical power. In addition, interviewer-administered 24-h dietary recalls can capture extensive and complete information on all foods and beverages consumed and provide a more precise assessment of the intake of wheat and rice. Further, mixed-effect modeling contributed to less biased, more precise estimates. Our study is not without limitations. A limitation to the study is the lack of separate data on whole grain and refined grain. Hence, we were not able to distinguish the association between whole grain from refined grain. However, whole grain is rarely consumed in China (46). Another limitation is that our analysis did not consider cooking or processed methods that might modify or confound the association of wheat and rice intake with certain health outcomes.

## Conclusions

In conclusion, we found that a high intake of wheat was positively associated with the risk of overweight/obesity among Chinese adults. Besides, we observed an inverse association between rice intake with overweight/obesity in Chinese men but not women. Given that dietary habits are becoming more westernized with a decrease in rice consumption and an increase in wheat consumption, the findings may have important public health implications for obesity prevention. On the other hand, it might be an important issue in nutritional education to draw attention to the benefits of rice consumption.

## Data Availability Statement

The original contributions presented in the study are included in the article/supplementary materials, further inquiries can be directed to the corresponding author.

## Ethics Statement

The studies involving human participants were reviewed and approved by the Institutional Review Committees of the University of North Carolina at Chapel Hill and the National Institute for Nutrition and Health, Chinese Center for Disease Control and Prevention. The participants provided their written informed consent to participate in this study.

## Author Contributions

JZ, ZW, WD, FH, and HW conducted data collection and data management. JZ and ZW conducted statistical analysis and interpretation. JZ conducted manuscript design and writing. BZ was primarily responsible for the supervision of the China Health and Nutrition Survey. HW had the primary responsibility for the final content. All authors contributed to the article and approved the submitted version.

## Funding

This research uses data from China Health and Nutrition Survey (CHNS). The CHNS receives grant funding from the National Institutes of Health (NIH) (R01-HD30880, DK056350, R24 HD050924, and R01-HD38700); the NIH Fogarty International Center (5D43TW007709 and 5D43TW009077) for financial support for the CHNS data collection and analysis files from 1989 to 2011; Carolina Population Center (5 R24 HD050924), University of North Carolina at Chapel Hill.

## Conflict of Interest

The authors declare that the research was conducted in the absence of any commercial or financial relationships that could be construed as a potential conflict of interest.

## Publisher's Note

All claims expressed in this article are solely those of the authors and do not necessarily represent those of their affiliated organizations, or those of the publisher, the editors and the reviewers. Any product that may be evaluated in this article, or claim that may be made by its manufacturer, is not guaranteed or endorsed by the publisher.
